# On-Chip Integration of Impedance Cytometry for Inline Optimization of Dielectrophoretic Separations on Multiple Cellular Biophysical Metrics

**DOI:** 10.1021/acssensors.5c00192

**Published:** 2025-06-06

**Authors:** Javad Jarmoshti, Abdullah-Bin Siddique, Aditya Rane, Alexandra R. Hyler, Sara Adair, Todd W. Bauer, Nathan S. Swami

**Affiliations:** † Electrical and Computer Engineering, 2358 University of Virginia, Charlottesville, Virginia 22904, United States; ‡ Chemistry, 2358 University of Virginia, Charlottesville, Virginia 22904, United States; § CytoRecovery, Inc., Blacksburg, Virginia 24060, United States; ∥ Surgery, School of Medicine, 2358 University of Virginia, Charlottesville, Virginia 22904, United States

**Keywords:** microfluidics, dielectrophoresis, impedance Cytometry, single-cell analysis, circulating tumor cells, automation

## Abstract

Microfluidic cell separation by dielectrophoresis, based on biophysical and electrical physiology metrics, is often optimized using on-chip fluorescence microscopy or off-chip flow cytometry of the separated fractions. However, these techniques require fluorescent reporters or stained samples that operate as end point assays, preventing the separated cell fractions from being utilized for longitudinal or transplantation studies. Single-cell impedance cytometry has a small footprint for facile integration toward label-free quantification of the separated fractions based on cell size, viability, and biophysical metrics. However, this is limited by low impedance signal-to-noise ratios in the low-conductivity media optimal for dielectrophoretic separation and by irreversible dielectrophoretic cell capture on impedance acquisition electrodes, while its single-cell resolution ability is limited by the high channel depths used to enhance sample throughput. Herein, using viscoelastic flows for elasto-inertial cell focusing over the channel depth, the throughput of dielectrophoretic separation is maintained, and bubble formation is avoided, while the downstream voltage for impedance cytometry can be maximized without irreversible cell capture to enhance impedance sensitivity in the dielectrophoretic separation media. This multichannel cytometry capability is integrated for automated optimization of the dielectrophoretic enrichment of live circulating tumor cells released into the suspension of pancreatic cancer cell cultures, using impedance metrics to monitor the separated fractions for feedback toward the selection of live cells within specific size ranges and with minimized transmembrane voltage-induced cell damage.

## Graphical abstract

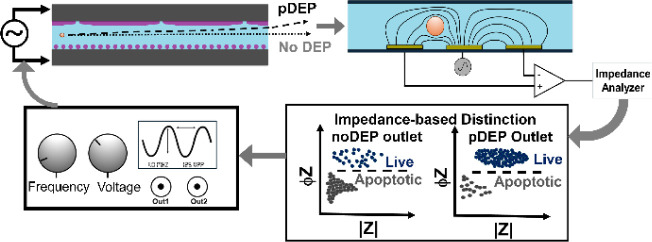

Phenotypic heterogeneity due to cell subpopulations^1^ within biologically relevant tumor, immune, and stem cell samples is an essential feature of cell types with multiple functions.^2^ The distinct cell functions can often be identified by their characteristic protein expression after binding to antibody receptors and fluorescent staining^3^ or magnetic functionalization^4^ for classification and sorting of cells by flow cytometry. However, identifying protein expression markers are unavailable in many systems due to the plasticity of metastatic cells,^5^ or for prediction of the differentiation lineage of stem cells^6^ or to quantify dynamics in immune cell activation.^7^ Label-free cellular separations^8^ based on their functionally relevant biophysical properties, such as size,^9^ shape,^10^ deformability^11^ and electrical physiology^12^ are emerging within microfluidic platforms, which can generate sufficient force fields for the specific deflection of a particular cell type from a heterogeneous sample. Microfluidic separations based on dielectrophoresis (DEP),^13,14^ wherein spatial electric field nonuniformities are designed for selective translation of polarized cells toward the high-field region by positive DEP (pDEP) or away from the high-field region by negative DEP (nDEP) due to polarization of the surrounding media^15,16^ are particularly significant for the enrichment of live *vs*. dead cells,^17^ circulating tumor cells,^18,19^ stem cell progenitors,^20^ cells based on their organelle phenotype,^21^ bacterial strain discrimination,^22,23^ and the isolation of cell-secreted extracellular vesicles.^24,25^

Sensors for inline monitoring of cell phenotype during microfluidic separations can be utilized for feedback to optimize the separation conditions through active control of separation force fields^26–29^ for the enrichment of a particular cellular composition or size distribution, thereby advancing automation,^30^ relaxing device design and flow rate requirements, and allowing for the application of versatile sample types. Currently, fluorescence microscopy is used to optimize microfluidic separations, but it requires stained cell samples that can only operate as an end point assay, wherein the separated cell fractions cannot be utilized within downstream longitudinal or transplantation studies. Label-free single-cell impedance cytometry,^31–33^ wherein cell electrical physiologies can be measured at high throughput (100–500 cells/s) based on their size distribution, viability, membrane capacitance, and cytoplasmic conductivity, is an ideal technique for on-chip integration toward inline monitoring of separations, since it offers multiparametric information and requires only a small device footprint,^34^ with no extra sample preparation steps. The integration of impedance cytometry downstream of microfluidic separation on cell size metrics has been presented using inertial focusing of neutrophil extracellular traps^7^ and circulating tumor cells,^35^ as well as in our work post-deterministic lateral displacement (DLD) separation for impedance-based monitoring of the enrichment of activated macrophages.^36^ However, the analogous integration of impedance cytometry downstream of DEP enrichment is more challenging. Since pDEP separations need to be conducted within media of low conductivity to maximize the dielectric contrast between the cell and its surrounding media for enhancing the DEP deflection force over drag forces on cells under continuous microfluidic flow, the lower media conductivity can lead to lower current signals during impedance cytometry. While the acquisition voltage for impedance cytometry can be increased to enhance the signal-to-noise ratio, the low conductivity media can cause irreversible dielectrophoretic cell capture on the impedance acquisition electrodes, thereby limiting its monitoring ability. While on-chip buffer swap^30^ post-DEP allows for impedance cytometry and impedance-activated DEP sorting,^37^ these can increase sample handling to cause cell loss within the separated fractions.

Viscoelastic fluids with cell samples immersed in synthetic polymers that exhibit Newtonian flow have been utilized at critical flow rates to control the 3D position of particles in the microchannel by initiating elastic lift force and inertial lift force.^38,39^ Our novelty is the utilization of such elasto-inertial focusing effects at moderate flow rates (1–10 μL/min) to position cell streamlines critically away from coplanar impedance acquisition electrodes, preventing irreversible DEP cell capture on one hand, while operating at flow rates that allow sufficient time for cells in the upstream dielectrophoretic separation field and sufficient sensitivity for downstream impedance measurement in low-conductivity media. This approach enables us to increase the impedance acquisition voltage to enhance the signal-to-noise ratio for cytometry measurements in DEP media of low conductivity, enabling its direct multichannel integration downstream of DEP separation. While prior work has demonstrated the utilization of viscoelastic flows with impedance cytometry in physiological media of high conductivity (1x-5x PBS) for elasto-inertial focusing to enhance event-to-event reproducibility,^40–42^ analogous studies in media of low conductivity (<0.01x PBS) are challenging due to their lower impedance signal-to-noise levels, the disruption of cytometry by irreversible DEP cell capture under the ensuing high dielectric contrast conditions, and the possible coupling of rheological and electrical properties of the carrier media.^43^ We address these challenges by optimizing the viscoelastic flow conditions to allow sufficient time for cell streamlines to be deflected by pDEP under sequential 3D field non-uniformities in the DEP separation stage, while maintaining cell streamlines in the downstream impedance cytometry stage to be at sufficiently high flow velocity and focused away from impedance acquisition electrodes to limit irreversible DEP-induced cell capture at the high acquisition voltages required for cytometry in low-conductivity media. This integrated platform is applied to monitor the dielectrophoretic enrichment of live circulating cancer cells that are released into the suspension of drug-treated pancreatic cancer cell cultures,^44^ which is of interest due to their resemblance to circulating tumor cells in terms of their ability to resist drug treatment and to survive in suspensions away from their extracellular matrix.^45,46^ Specifically, we demonstrate the ability of on-chip impedance cytometry to monitor the separated cell fractions for feedback over multiple metrics to optimize the DEP conditions (voltage, frequency, and media conductivity) for cell enrichment based on metrics of viability, size distribution, and lowered cell damage due to transmembrane potential drop that occurs near the DEP crossover frequency.^47^

## Results and Discussion

### Device Design and Integration

Using the single-layer (50 μm depth) polydimethylsiloxane (PDMS) device per Figure 1A, viscoelastic sample and sheath flows of cells immersed in poly­(ethylene oxide) (0.6 MDa PEO) focus the incoming cell streamline (net flow rates of 1–10 μL/min for Reynolds numbers in the 0.1–1 range) toward the center and displaced along the orifices of the 100 μm wide channel that forms the DEP separation stage (Figure 1B) to cause the pDEP deflection of specific cell streamlines. This occurs due to voltage across 3D Field’s metal electrodes patterned at the sample channel wall edges and sequential nonuniformities due to orifices with 20 μm openings to create high-field regions on one side, and rounded posts for capillary pinning of the Field’s metal for a flattened profile to create low-field regions on the other side of the sample channel.^48^ The undeflected (so-called “noDEP”) and pDEP-deflected (“pDEP”) streamlines are designed to be routed to different outlets that each feature a constricted region of 50 μm width and depth to increase flow velocities. In these outlets, three coplanar gold electrodes (25 μm width and 15 μm spacing) are patterned on a glass coverslip and aligned orthogonal to the PDMS channel, prior to bonding and electrical wiring per Figure 1C. The impedance amplitude (|Z|) and phase (ϕZ) are continuously acquired during the entire DEP separation and plotted at the optimal frequency that enables the distinction of live versus dead or apoptotic cells in the DEP media of low conductivity (Figure 1D). Based on thresholds for |Z| and ϕZ under optimal signal acquisition conditions (described subsequently), a MATLAB code is utilized to trigger the DEP amplifier from its serial port, so that the DEP excitation voltage and frequency can be varied to optimize the live cell numbers of specific size ranges in the pDEP-separated fraction (Figure 1E,F). This enables closed-loop operation, wherein impedance-based monitoring of the target cells within the separated fraction is utilized for automated optimization of DEP separation conditions, thereby maximizing collection efficiency and sample purity from heterogeneous and unknown starting samples.

**Figure 1:**
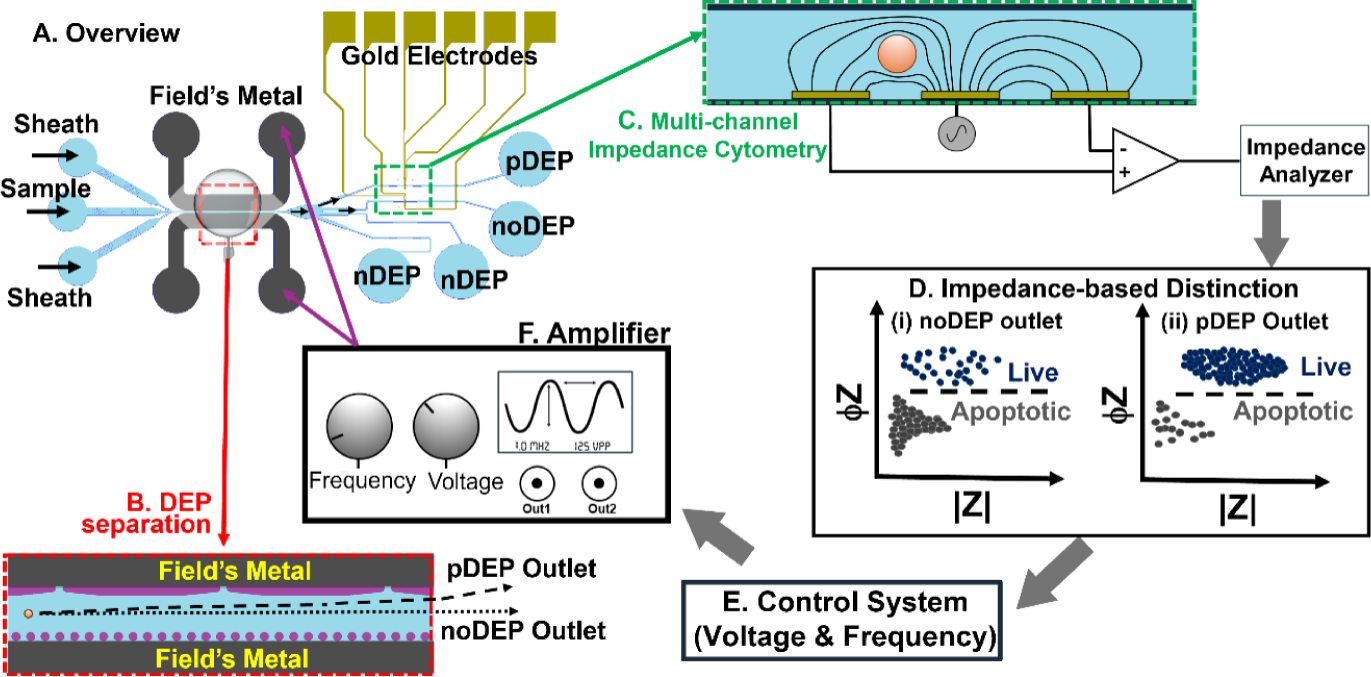
A. Device overview for integrating multichannel impedance cytometry to monitor dielectrophoretic (DEP) separated fractions; B. DEP separation showing the deflected positive DEP (pDEP) and the undeflected (noDEP) streamlines due to voltage across 3D Field’s metal electrodes patterned at the sample channel wall edges and from field nonuniformities due to orifices (high field) on one side and posts (low field) on the other side of sample channel. C. Coplanar electrodes with their electrical wiring for single-cell impedance cytometry. D. Schematic for gating live vs apoptotic cells based on their impedance phase (ϕZ) and magnitude (|Z| or cell electrical size = $\sqrt[{3}]{{|Z|}_{0.05\mathrm{MHz}}}$ ). Based on thresholds for |Z| and ϕZ, a MATLAB code triggers the DEP amplifier from its serial port (E), so that DEP excitation voltage and frequency can be varied (F) to optimize for live cell numbers of specific size ranges in the pDEP separated fraction. Such integrated on-chip monitoring approaches to quantify separated cell fractions can advance active operational control by tuning for the selection of specific cell phenotypes from versatile sample sets.

### Reducing DEP-Induced Cell Capture on Electrodes

The enrichment of live cells by pDEP requires low-conductivity media (σ_med_) that enable a high level of dielectric contrast between cells and the media, as apparent from simulations of the Clausius-Mossotti factor (Re­(f_CM_) in Figure 2A), which reaches its maximum level (unity) over a wide frequency range (0.3–10 MHz) in σ_med_ of 55–100 μS/cm. While the device in Figure 1 can be wired to also monitor the pDEP and nDEP-separated fractions by impedance cytometry, significant nDEP levels for dead cells can only be reached in media of higher conductivity (>1000 μS/cm), which is impractical since it reduces the pDEP level for live cells (Figure 2A). Hence, the nDEP channels of Figure 1 are not used directly for cell collection but for balancing flow resistances to enable effective collection of live cells in the pDEP outlet and dead cells in the “no DEP” outlet. The impedance signal sensitivity within the lowered σ_med_ is assessed using electric field simulations (Figures S1A and 2B) and impedance measurements (Figures S1B and 2C).

**Figure 2:**
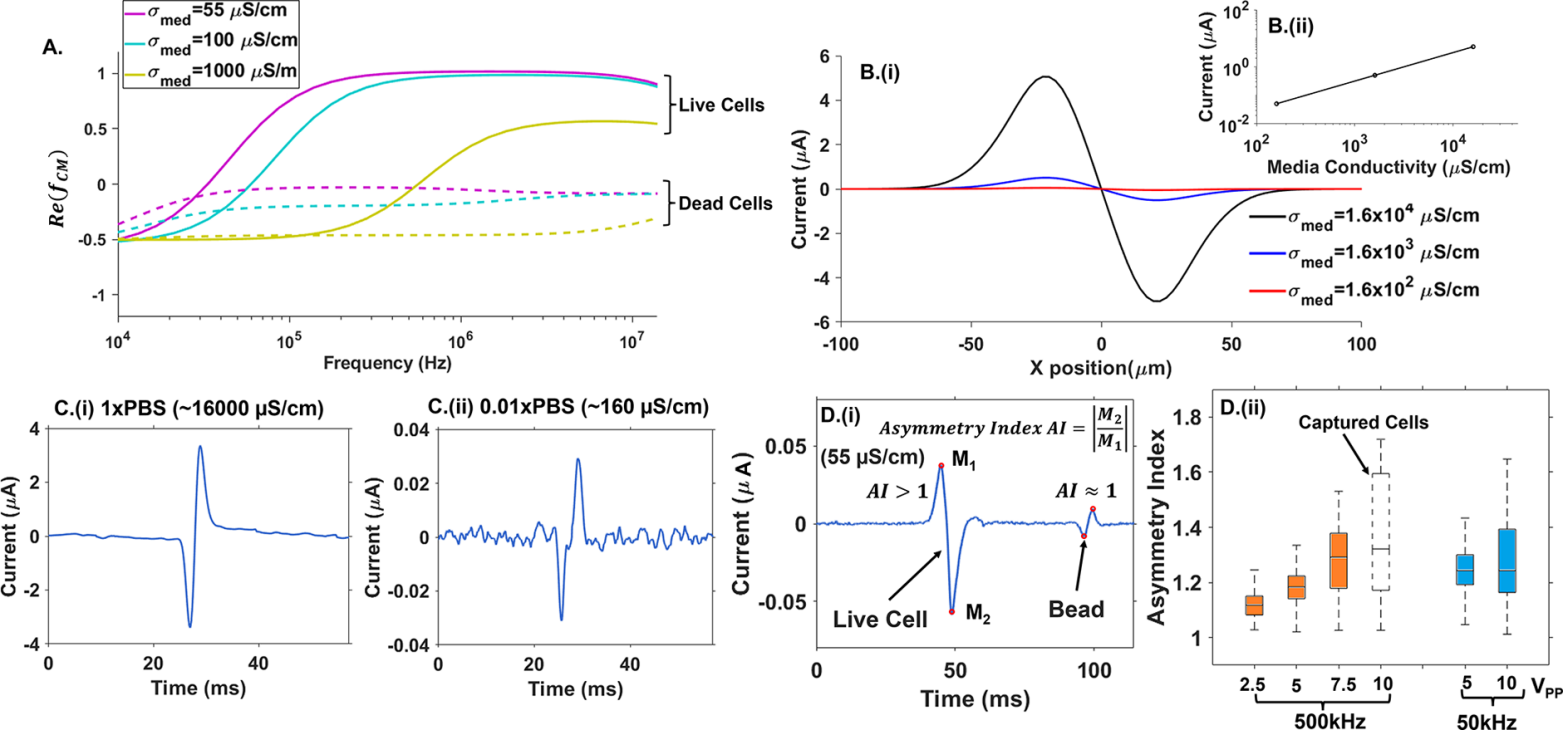
A. Dielectrophoretic enrichment of live cells requires media of low conductivity (σ_med_), as apparent from the higher level of the Clausius-Mossotti factor (Re­(*f*_*CM*_)) in σ_med_ of 55–100 μS/cm. Impedance cytometry in progressively lower σ_med_ using signal simulations (B) and measurements (C–D). B. Simulations of the bipolar Gaussian impedance signal show that a 100-fold drop in σ_med_ causes ∼ 100-fold drop in signal (5 to 0.03 μA), which remains within signal sensitivity limits. C. Optimized measurements show similar impedance signal sensitivity to σ_med_ drop (3.5 μA (i) to 0.03 μA (ii) for 100-fold drop in σ_med_), with sufficient signal-to-noise level for detection of 12 μm beads in the low σ_med_. D. (i) The asymmetry index of the bipolar Gaussian shape (*AI*) is used to (ii) optimize the impedance acquisition voltage and frequency for detection of live cells at sufficient signal-to-noise in low σ_med_ (55 μS/cm), while reducing signal distortion due to irreversible cell capture by DEP.

Simulations of the bipolar Gaussian impedance signal (Figure 2B) show that a 100-fold drop in σ_med_ causes an ∼100-fold drop in signal (5 to 0.03 μA), with measurements demonstrating similar levels of signal sensitivity to σ_med_ (Figure 2C­(i)-(ii)). To optimize the impedance signal-to-noise levels for the detection of cells in low σ_med_ (55 μS/cm), the acquisition voltage is increased (2.5 to 10 V_pp_) at two frequencies (500 and 50 kHz) after using viscoelastic flow (∼5 μL/min) for elasto-inertial focusing. Impedance-based electrical size ( $\sqrt[{3}]{{|Z|}_{0.05\mathrm{MHz}}}$ ) data (Figure S1C) on 12 μm polystyrene beads in the absence of viscoelastic flow show that the characteristic depth-focusing points within a rectangular channel cause two distinctive data clusters in the electrical size histogram, which merge under viscoelastic flow (1–10 μL/min) to form a single data cluster of narrow width (12 ± 1 μm), suggesting well-focused beads. In this manner, cell streamlines are focused over a critical depth range to be sufficiently away from the coplanar impedance acquisition electrodes to prevent irreversible DEP capture while maintaining them within a depth range that is sufficient for obtaining single-event sensitivity to measure field screening in low σ_med_. While higher acquisition voltages enhance impedance signal-to-noise levels, the ensuing higher DEP forces in low σ_med_ (55–160 μS/cm), as apparent from the higher Re­(f_CM_) in Figure 2A at frequencies ≥ 50 kHz, could lead to DEP-induced bubbles and alteration of cell streamlines, distorting the bipolar Gaussian signal shape. Cell capture by DEP at the latter set of impedance acquisition electrodes (Figure S2A shows an example image and Movie S1) alters the background impedance level measured at the first set versus second set of electrodes. This would disrupt one side of the temporal impedance signal profile versus the other side, thereby distorting the differential signal shape and its background level under cell capture (Figure S2B,C). Using the asymmetry index or *AI* of the bipolar Gaussian signal shape under differential signal acquisition (Figure 2D­(i)) to detect DEP-induced bubbles and distortions that culminate in cell capture, the data acquisition voltage (2.5 to 10 V_pp_) and frequency (500 and 50 kHz) are optimized. Per Figure 2D­(ii), the 500 kHz level that is typically used in 1x PBS media to quantify the electrical field screening of cells leads to a sharp rise in signal distortion at higher voltages in low σ_med_ of 55 μS/cm, due to DEP-induced alteration of cell streamlines, with DEP capture at 10 V_pp_ altering the signal backgrounds (Figure S2C). On the other hand, at impedance acquisition frequencies of 50 kHz in σ_med_ of 55 μS/cm, the increase in acquisition voltage (5–10 V_pp_) causes a less sharp rise in signal asymmetry, with no signal background alteration due to DEP cell capture. Hence, we infer that viscoelastic flow (0.5% w/w of 0.6 MDa PEO at 5 μL/min) enables elasto-inertial cell focusing within critical depth ranges in the microchannel to facilitate impedance measurements in low σ_med_ (55–160 μS/cm) at acquisition voltages up to 10 V_pp_ for optimizing signal sensitivity while avoiding DEP-induced cell capture on the impedance acquisition electrodes.

### Impedance-Based Live vs Dead Cell Detection in Low σ_med_

Using simulations and measurements, we ascertain impedance-based distinction of live *vs*. dead cells in the low σ_med_ (55–160 μS/cm) that is required for pDEP enrichment of live cells, especially at the low impedance acquisition frequency levels (50 kHz per Figure 2D­(ii)) needed to avoid DEP-induced cell capture on electrodes. Simulations of the electrical field screening in low σ_med_ (0.01x PBS) show that the fields penetrate live cells at 50 kHz (Figure 3A­(i)) and to a greater extent at 20 MHz (Figure 3A­(ii)). On the other hand, fields are predominantly screened around dead cells, with minimal penetration at 50 kHz (Figure 3B­(i)), while some field penetration occurs at 20 MHz (Figure 3B­(ii)), albeit below that for live cells. Comparison of field screening at 20 MHz to that at 50 kHz can potentially distinguish live vs dead cells; however, frequency levels ≥ 0.5 MHz cause irreversible cell capture by pDEP on the impedance electrodes (Figure 2D­(ii)). Using only 50 kHz frequencies for cytometry, the field lines that penetrate live cells (Figure 3A­(i)) due to polarization (σ_cell_ > σ_med_) are reversed to become predominantly screened for dead cells (Figure 3A­(ii)) due to inverse polarization (σ_cell_ < σ_med_), suggesting that live–dead distinction is possible. Measured impedance cytometry signals in low σ_med_ (160 μS/cm) at 50 kHz show that live cells can be detected well above the noise level (Figure 3C), with the real and imaginary parts of their differential signal indicating field penetration (positive signal for cell presence and negative signal for cell absence between electrodes), per simulations (Figure S3). On the other hand, while dead cells can be detected just above the noise level (Figure 3D), the net differential signal shape is reversed (negative preceding positive) to suggest field screening, in agreement with the simulations (Figure 3A,B).

**Figure 3:**
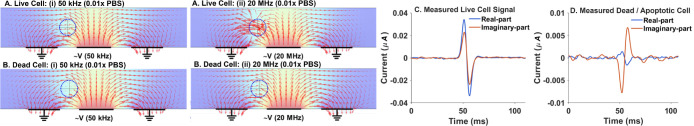
A. Electric field screening simulations of (A) live, and (B) dead cells (dotted circles) in 0.01x PBS (low σ_med_) at 50 kHz (i) and 20 MHz (ii). The high level of field penetration to the interior of live cells (σ_cell_ > σ_med_) is modified for dead cells (σ_cell_ < σ_med_) to field screening with minimal penetration at 50 kHz and greater penetration at 20 MHz, albeit below the respective level for live cells. Measured impedance cytometry differential signals at 50 kHz in σ_med_ of 160 μS/cm for: (C) live cells show that high field penetration causes positive signal at early transit time versus negative signal at later transit times, (D) dead cells show a reversal in differential signal shapes over transit time due to field screening under inverse polarization.

### Validating On-Chip Impedance Cytometry with Off-Chip Flow Cytometry

To validate the integration of impedance cytometry for inline monitoring of the DEP-separated fractions, we consider samples obtained from cultures of patient-derived metastatic pancreatic cancer cells^49^ (MAD-608) from pancreatic ductal adenocarcinoma (PDAC) that are enlarged in xenograft models and express GFP reporters. According to the flow cytometry results (Figure 4A­(i)), untreated adhered PDAC cultures show 65% live (GFP+) and 35% dead cells (GFP-). Drug treatment (1 μg/mL gemcitabine for 48 h) leads to ∼88% dead cells in the suspension (GFP-) per Figure 4A­(ii). Using equal proportions of untreated and drug-treated PDAC samples, a mixed sample is constituted with 43% live cells (GFP+) and 57% dead cells (GFP-) (Figure 4A­(iii)) that is subsequently used to quantify the enrichment of live PDAC cells based on downstream on-chip impedance cytometry. Using flow cytometry on this mixed sample, we validate the similarity in live *vs.* dead cell percentage levels obtained using GFP reporters (Figure 4B­(i)) to those from a standard live–dead stain (Figure 4B­(ii) using ZNIR or Zombie near-IR stain for dead cells. Hence, the GFP level can be used for off-chip validation of live cell events that are measured by on-chip impedance cytometry. The impedance cytometry data of the respective samples from Figure 4A­(i-iii) measured at 50 kHz in σ_med_ of 160 μS/cm are plotted as ϕZ vs electrical size or $\sqrt[{3}]{{|Z|}_{0.05\mathrm{MHz}}}$ (Figure 4C­(i-iii)) after data normalization against co-flowing polystyrene beads of 12 μm size to accurately reflect the |Z| levels, while the ϕZ levels are normalized to zero for the insulating beads. Based on this, live cell events within the respective samples exhibit positive ϕZ levels due to field penetration through the cell caused by dielectric polarization at the cell surface (σ_cell_ > σ_med_). Dead cell events, on the other hand, exhibit negative ϕZ levels due to field screening by the cell caused by dielectric polarization at the media interface to the cell (σ_cell_ < σ_med_). Furthermore, based on |Z|, live cell events exhibit larger electrical sizes, whereas dead cell events exhibit significantly smaller sizes, indicating that cell shrinkage from apoptosis is the primary cell death pathway^50^ (see correspondence of electrical size to physical cell size in Figure S4). Hence, Figure 4C­(i-iii) shows facile gating of live *vs*. dead or apoptotic cells on ϕZ and |Z| metrics based on live cells showing positive ϕZ levels, and dead cells showing negative ϕZ levels with smaller electrical size *vs*. live cells. On-chip impedance cytometry indicates higher event numbers for the majority population versus off-chip flow cytometry, in terms of live cells in the untreated sample Figure 4C­(i) and dead cells in the drug-treated sample Figure 4C­(ii). It is likely that sample transfer for off-chip flow cytometry leads to viability losses in the majority live cells of the untreated sample and systematic loss of the majority smaller-sized dead cells in the drug-treated sample, while on-chip impedance cytometry avoids these losses. The mixed sample compares well between the two cytometry systems (Figure 4A­(iii) *vs*. [Fig fig4]C­(iii)), thereby confirming minimal effect of the PEO media used for on-chip impedance cytometry on cell viability (also see Figure S5).

**Figure 4:**
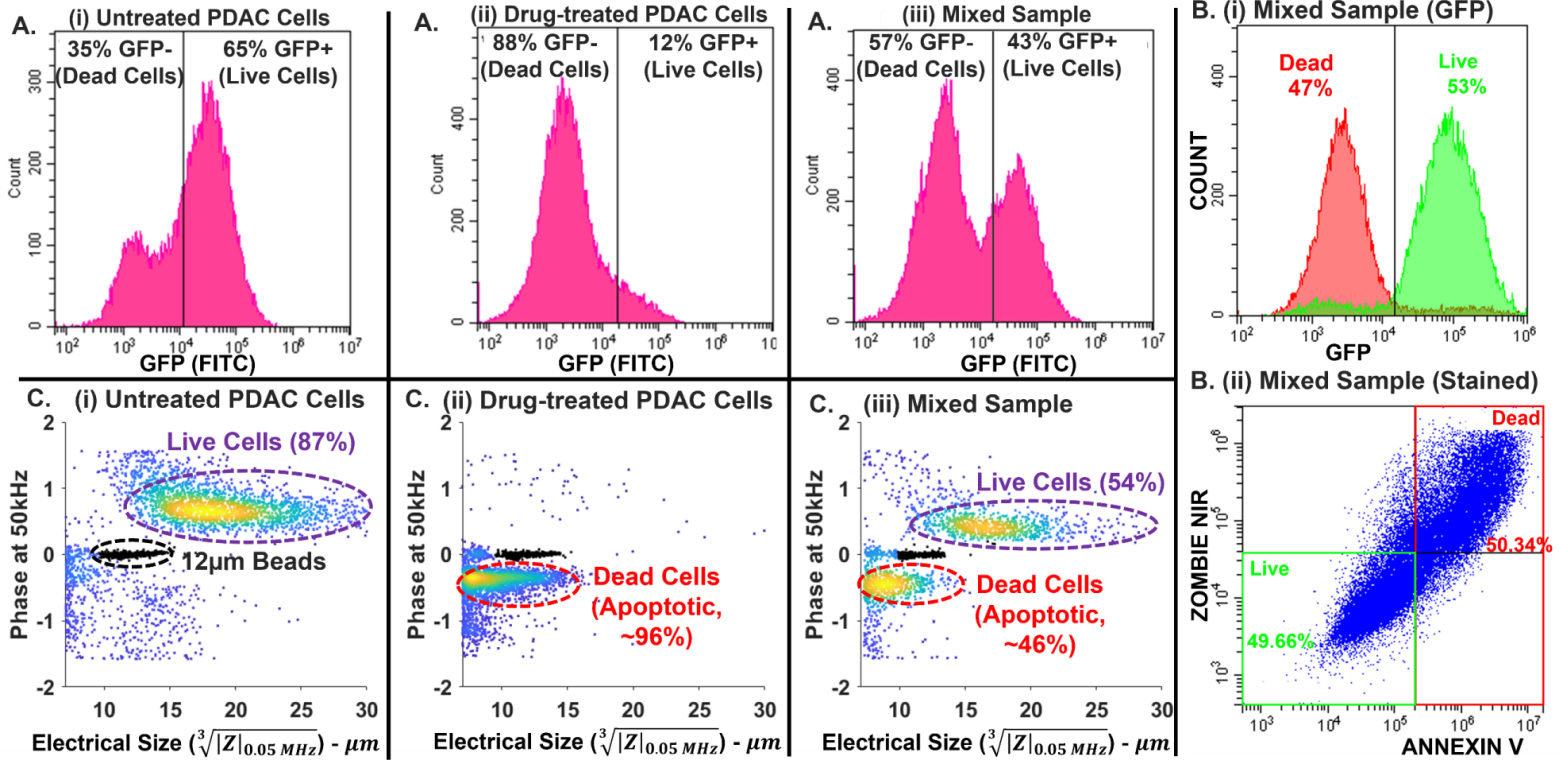
A. Sample types used for enrichment of live pancreatic cancer (PDAC) cells. (i) Predominantly live cells (65%) from untreated adhered PDAC cultures; (ii) Predominantly dead cells (88%) from the suspension of drug-treated PDAC cultures; (iii) Mixed samples of (i) and (ii) (43% live and 57% dead) measured by flow cytometry based on their GFP signal. B. The GFP signal expressed by live cells is lost for dead cells, as confirmed by GFP flow cytometry of the mixed sample (i) and validated by live–dead staining (ii). C. Impedance cytometry of the respective samples from A­(i-iii) at 50 kHz in σ_med_ of 160 μS/cm shows facile gating of live *vs*. dead or apoptotic cells on ϕZ and |Z| metrics, with live cells showing positive ϕZ levels and dead cells showing negative ϕZ levels and smaller electrical size ( $\sqrt[{3}]{{|Z|}_{0.05\mathrm{MHz}}}$ ) vs live cells, which indicates cell death by apoptosis.

### On-Chip Cytometry to Optimize pDEP Separation

Utilizing these |Z| and ϕZ levels to differentiate live vs apoptotic PDAC cells, the conditions for pDEP enrichment of live cells from the mixed PDAC sample are optimized. Specifically, on-chip impedance cytometry downstream of pDEP enrichment in σ_med_ of 55 μS/cm is used to optimize the voltage for pDEP separation. In the absence of the DEP separation field (0 V_pp_), the input sample passes fully into the undeflected “noDEP” outlet based on events in the live cell data cluster in Figure 5A­(i), with no live or dead cell events in the deflected “pDEP” outlet (Figure 5A­(ii)), thereby confirming that the focusing flow near the input maintains focused cell streamlines over the ∼5 mm length of the DEP separation stage, with no flow dispersion into the pDEP outlet. Our prior work on DEP separation of live *vs.* dead PDAC cells^44^ using similar devices and mixed samples optimized the enrichment of live cells at the DEP excitation of 25 V_pp_ at 1 MHz. In the current work, however, due to the need for viscoelastic flows for elasto-inertial focusing of cell streamlines away from the impedance acquisition electrodes to prevent their DEP capture, higher DEP excitation voltages (100–200 V_pp_) are required in the separation stage to ensure sufficient DEP deflection force over the drag forces. Per eq S11, the DEP mobility (μ_DEP_) is lowered with increasing viscosity (η), thereby requiring greater ∇*E* ^2^ for equivalent velocities of cells under the balance of DEP and viscoelastic drag forces, which is addressed by higher voltages for DEP. To reduce the incidence of cell damage at this higher voltage due to transmembrane potential drop that can occur near the DEP crossover frequency, DEP excitation frequencies of 1–5 MHz that are well above the DEP crossover frequency at this σ_med_ of 55 μS/cm (Figure 2A) are utilized. As the voltage for DEP is gradually increased at 1 MHz in the 100–150 V_pp_ range, the impedance data clusters from cell fractions in the noDEP (Figure 5B,C­(i)) and pDEP outlets (Figure 5B-D­(ii)) show progressively greater deflection of live cells into the pDEP outlet, with substantially fewer live cell events remaining in the noDEP outlet (see flow cytometry in Figure S6). At the DEP excitation of 200 V_pp_ at 1 MHz, very few live cell events remain in the noDEP outlet (Figure 5D­(i)), with the pDEP outlet showing only live cell events and no apoptotic cell events (Figure 5D­(ii)). The collection efficiency is computed based on the proportion of live cell events in the pDEP outlet to the total live cell events in the noDEP and pDEP outlets. From Figure 5E, it is apparent that the collection efficiency from on-chip impedance cytometry is comparable to the levels obtained based on the GFP signal from off-chip flow cytometry, validating the use of on-chip cytometry for the DEP monitoring.

**Figure 5:**
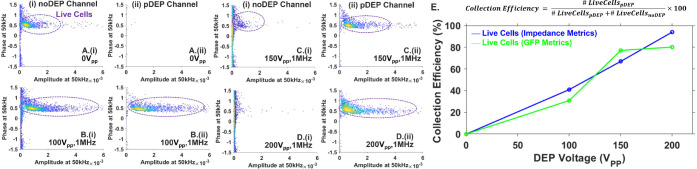
On-chip impedance cytometry for inline optimization of DEP voltage at 1 MHz in σ_med_ of 55 μS/cm to enhance collection efficiency (defined in E) at: A. 0 V_pp_; B. 100 V_pp_; C. 150 V_pp_; D. 200 V_pp_. E. Collection efficiency at various DEP voltages obtained from on-chip impedance cytometry (|Z| and ϕZ metrics) resembles that from GFP levels by off-chip flow cytometry. Live cell #s in “noDEP” and after pDEP deflection is based on live cell GFP thresholds of Figure 4B and |Z| and ϕZ metrics of Figure 4C.

### On-Chip Cytometry to Monitor DEP-Induced Cell Damage

Next, we explore the ability of on-chip impedance cytometry to monitor DEP-induced cell damage due to transmembrane potential drop. Such electroporation can occur under DEP excitation within higher σ_med_ than the DEP at lower σ_med_ (55–160 μS/cm), due to the greater current flow from the media into the cell that enhances cell damage due to transmembrane potential drop. The DEP-induced cell damage can be monitored based on ϕZ and |Z| levels using downstream on-chip impedance cytometry, enabling feedback to alter the DEP excitation to reduce cell damage. Using the mixed PDAC sample in σ_med_ of 220 μS/cm at DEP excitation levels of 100 V_pp_ at 1 MHz, the undeflected noDEP fraction chiefly includes apoptotic cells (Figure 6A­(i)), but some live cells also pass through undeflected. The live cells deflected into the pDEP fraction (Figure 6A­(ii)) show no discernible alterations in the ϕZ and |Z| levels compared to live cells in the input samples (Figure 4C­(iii)). At higher DEP excitation levels of 150 V_pp_ at 1 MHz in σ_med_ of 220 μS/cm, the pDEP fraction exhibits one data cluster for live cells at positive ϕZ levels and another data cluster at negative ϕZ levels, which we term electroporated cells (Figure 6B­(ii)). While the negative ϕZ levels of the latter data cluster indicate their inability to electrically polarize due to cell damage, their high |Z| levels indicate that these electroporated cells are closer in electrical size resemblance to live cells and differ from the smaller-sized apoptotic cells (Figure 6B­(i)), suggesting that they likely originated from the live cells. By establishing thresholds in ϕZ and |Z| levels to gate for live, apoptotic, and electroporated PDAC cells, we show based on the pDEP collection efficiency plot in Figure 6C that analogous DEP excitations of 150–200 V_pp_ at 1–2 MHz do not lead to any detectable cell damage at lower σ_med_ levels of 55 μS/cm. In this manner, downstream impedance cytometry enables monitoring of electrically induced cell damage within cell events of the pDEP fraction, so that the DEP excitation conditions can be altered to reduce cell damage.

**Figure 6:**
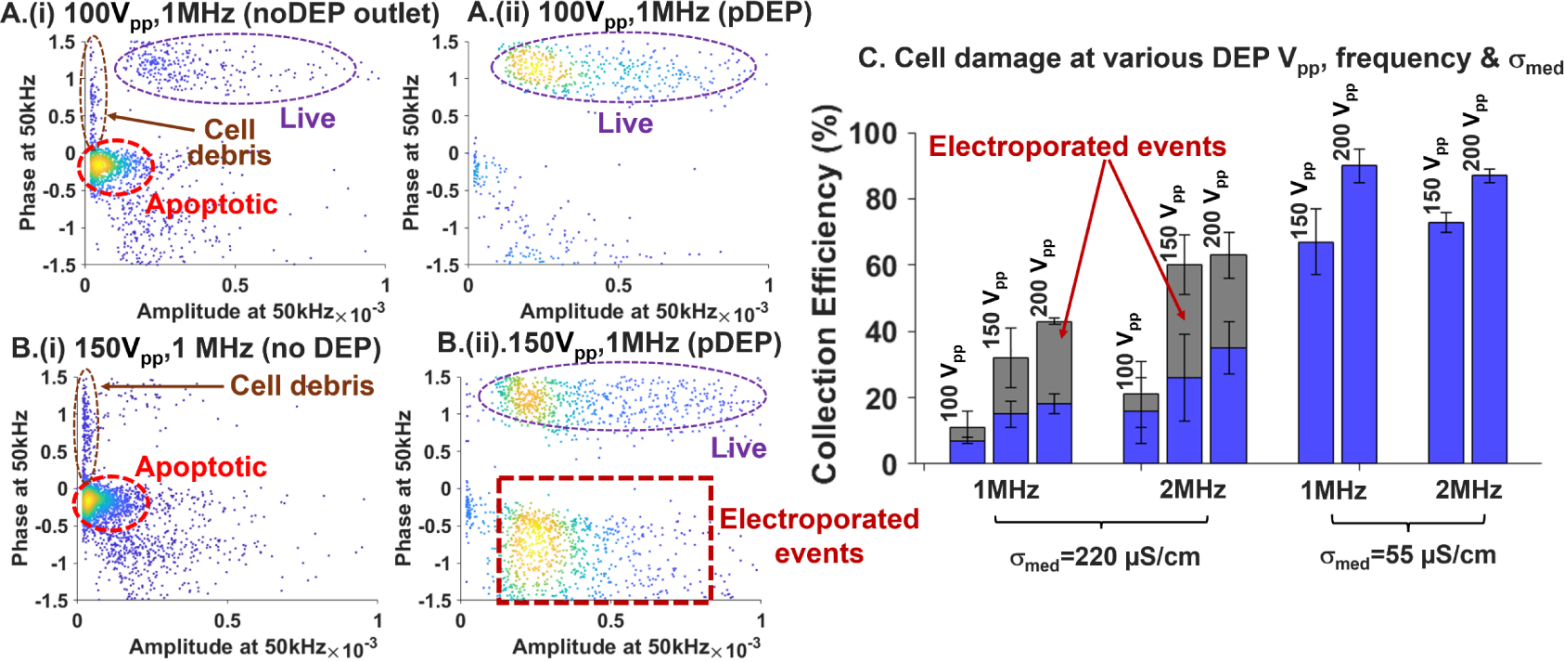
On-chip impedance cytometry for inline assessment of DEP field effects on cells in higher σ_med_ of 220 μS/cm (A and B) vs lower σ_med_ of 55 μS/cm (C) at different DEP excitation voltages and frequencies. Using σ_med_ = 220 μS/cm at: A. 100 V_pp_ and B. 150 V_pp_ at 1 MHz, cytometry plots shown in noDEP (i) and pDEP (ii) outlets. B.(ii) Electroporated or electric field induced cell pores occur at higher σ_med_ cause ϕZ to shift to negative levels, while maintaining |Z| or electrical size at the same level, distinguishing these from live cells (positive ϕZ and high |Z|) and apoptotic cells (negative ϕZ and low |Z|). C. DEP collection efficiency to optimize the DEP voltages, frequencies and σ_med_ that maximize live cells enrichment with no electroporation.

### On-Chip Cytometry to Size-Select Live Cells by DEP Enrichment

Since biological samples are heterogeneous across multiple metrics, it is often of interest to select cells based on more than one metric, such as the size distribution range of cells with a specific phenotype (e.g., live *vs*. dead cancer cells or activated *vs*. naïve immune cells). Based on the frequency dispersion of *f*_*CM*_ (Clausius-Mossotti factor) (Figure 2A), the lower levels of *Re*(*f*_*CM*_) at DEP frequencies in the vicinity of the crossover to pDEP (≤1 MHz) lead to its predominant dependence on cell size effects, due to their volumetric contribution to the pDEP trapping force. On the other hand, at frequencies far higher than the crossover (1–10 MHz), the *Re*(*f*_*CM*_) reaches its maximum value (unity), thereby minimizing the size dependence of the pDEP-enriched fraction. In this manner, through tuning the DEP excitation frequency and σ_med_, it is possible to size-select cells of a specific phenotype (e.g., live *vs*. dead cells). Since on-chip impedance cytometry can classify the DEP-separated fractions based on cell size and phenotype (e.g., viability), it enables real-time feedback to tune DEP excitation conditions for enriching size-selected cells of a specific phenotype.

Using the mixed PDAC sample in σ_med_ of 55 μS/cm at the DEP excitation level of 150 V_pp_, the DEP frequency is varied (1–5 MHz) to assess the size selection of live cells. Based on ϕZ *vs*. |Z| from on-chip impedance cytometry of the pDEP fraction (Figure 7A-C), successively higher frequencies increase the number of live cell events, as apparent from the progressively greater collection efficiency obtained at higher DEP frequencies (Figure 7D). Interestingly, in comparison to the broad size distribution of the input sample (Figure 7E­(i)), on-chip impedance monitoring of the pDEP-enriched fraction at 1 MHz shows a narrower size range composed chiefly of larger-sized cells that are gradually downshifted in average size and broadened in size distribution as DEP excitation increases toward 5 MHz (Figure 7E­(ii)), thereby causing live cell enrichment over the full size range of the input sample (smaller and larger-sized cells). This is also apparent based on the greater number of live cell events of lower |Z| in Figure 7C (5 MHz) *vs.* Figure 7A,B (1–2 MHz). Hence, on-chip impedance cytometry indicates that DEP excitation at 1–2 MHz can be used to size-select in a narrower cell size range, while DEP excitation at 5 MHz can be used to add increasing numbers of the smaller-sized cells from the input sample into the pDEP channel, with thresholds for ϕZ (live vs dead cells) and |Z| (large vs small cell sizes) used for feedback to optimize the DEP excitation frequency for size selection of live cells.

**Figure 7:**
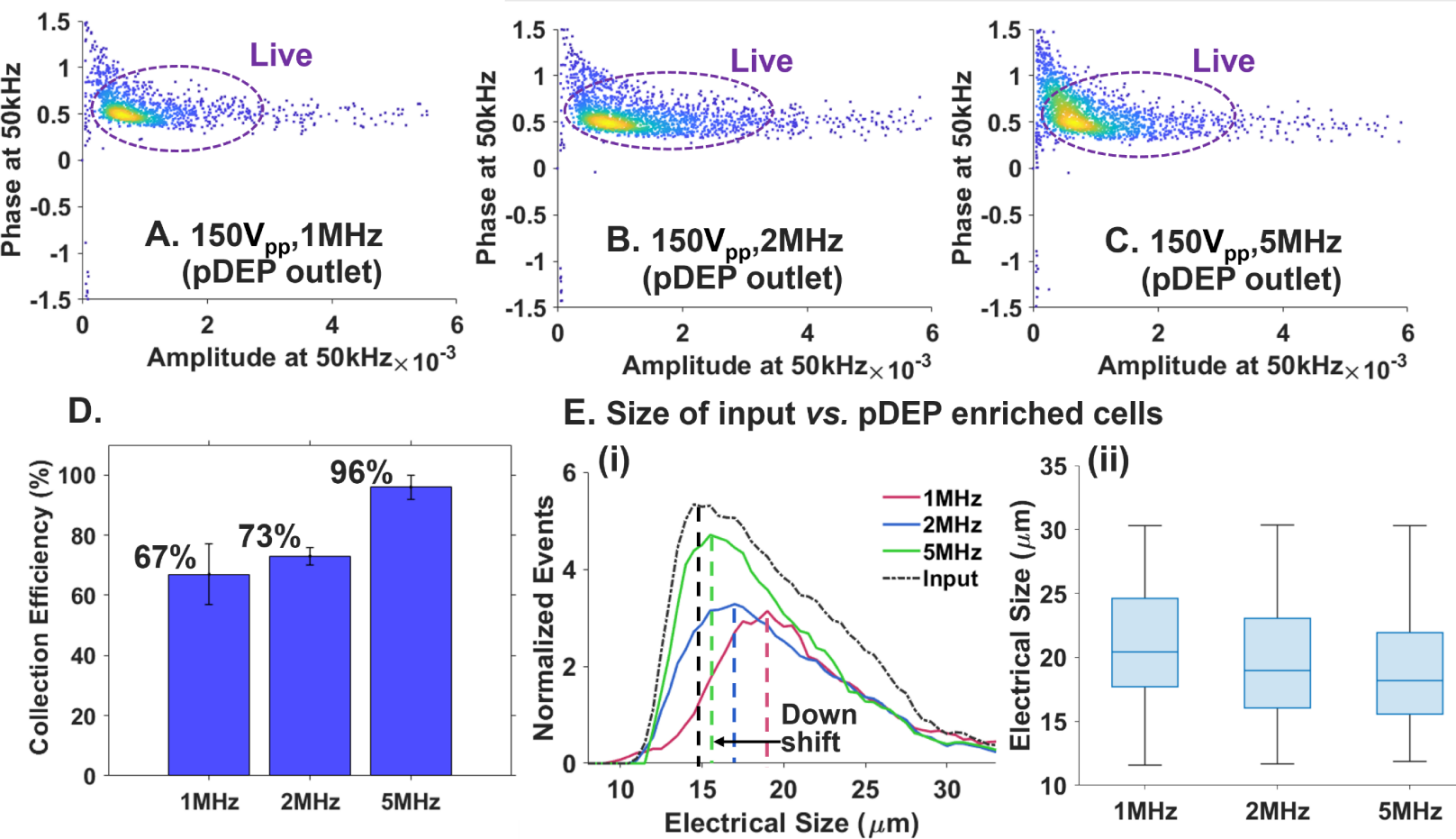
On-chip impedance cytometry for size-selection of live PDAC cells through inline optimization of pDEP excitation (150 V_pp_) frequency: A. One MHz; B. Two MHz and C. Five MHz; using ϕZ and |Z| metrics to select live cells of specific size ranges in pDEP fraction. D. pDEP collection efficiency, and: E. respective cell size distributions (i), with averages in box plot (ii).

## Conclusions and Outlook

On-chip integration of cytometry to measure microfluidic-separated fractions can allow inline control of separations to generate samples that are enriched across specific sets of cellular biophysical metrics (e.g., cell viability, electroporation, and size distribution), while avoiding biased sample loss and cross-contamination at the collection outlets that often occur during off-chip cytometry. Due to the small device footprint of impedance cytometry, the need for minimal sample preparation, and its ability to characterize cell phenotypes across multiple biophysical metrics, we integrate it downstream of dielectrophoretic separations. However, using impedance cytometry to distinguish live *vs*. apoptotic cells from heterogeneous samples with a wide size distribution is challenging in the low-conductivity media needed for dielectrophoretic separations due to its lower signal-to-noise ratio and irreversible DEP cell capture on acquisition electrodes at high voltages. We address this challenge by using viscoelastic flows for elasto-inertial cell focusing within a critical depth range that maintains DEP separation throughput while localizing cells away from the acquisition electrodes to enable voltage modulation to enhance impedance signal-to-noise ratios. In this manner, we optimize the viscoelastic flow (cells in 0.5% w/w of 0.6 MDa PEO at 1–10 μL/min) and impedance acquisition frequencies (50 kHz in low σ_med_ of 55–220 μS/cm) for setting thresholds in ϕZ (positive for live *vs*. negative for apoptotic cells) and |Z| (broad cell size distribution for live cells *vs*. narrow size distribution of small size for apoptotic cells) for single-cell phenotypic distinction by normalization against co-flowing polystyrene beads. These impedance signal metrics are utilized for inline control of DEP separation conditions, such as voltage, frequency, and media conductivity to optimize for the collection efficiency of live cells within specific size ranges and with minimal cell damage due to transmembrane potential effects that occur in higher σ_med_ and closer to the DEP crossover frequency. Future work will focus on impedance cytometry for monitoring and control of cell separations along alternate phenotypic metrics, such as the activation state of immune cells, differentiation lineage of stem cells, and metastatic potential of cancer cells within specific size cutoffs to generate enriched live cell samples.

## Materials and Methods

### Cell Sources

PDAC tumor samples were generated from specimens collected in collaboration with the University of Virginia Biorepository and Tissue Research Facility after approval of the University of Virginia Institutional Review Board for Health Sciences Research and after informed written consent from patients. MAD-T608 tumors were propagated in the pancreas of immunocompromised mice. Xenograft lines were established, transfected with GFP, selected using puromycin, and maintained in RPMI 1640 with 10% FBS and 2 mM glutamine.

### Cell Sample Generation

GFP-transfected tumor cells were plated at 4 × 10^5^ cells/mL in 6-well plates and allowed to adhere overnight in complete media (RPMI + 10% FBS). The drug-treated sample was generated by gemcitabine treatment of the plates at a concentration of 1 μg/mL for 48 h, with floating cells collected from the media supernatant in the wells after centrifugation (300g for 10 min). The untreated sample was generated by washing the adherent cells from the plates in 1× PBS (Thermo Fisher), followed by 0.05% trypsin treatment (Thermo Fisher) for 10 min, with added complete media for centrifugation (300g for 10 min). The mixed sample was generated from equal proportions of the untreated and drug-treated samples. Cell pellets of each sample type were resuspended in 1 mL of DEP buffer (sucrose and BSA, or bovine serum albumin, at a media conductivity of 55–220 μS/cm) and counted using a hemocytometer. These cell samples were utilized for dielectrophoretic (DEP) enrichment with downstream impedance cytometry.

### Device Design and Fabrication

The integrated device (Figure 1A) consists of a DEP separation stage (Figure 1B) and an impedance cytometry stage (Figure 1C). For the DEP separation stage (Figure 1B), viscoelastic flow (0.5% w/w of 0.6 MDa PEO) from two sheath inlets was used to focus the cell sample, which was also immersed in viscoelastic media. The active region of the separation stage consists of a microfluidic channel (1 mm length and 100 μm width) flanked by two electrode channels that adjoin sequential orifices (20 μm) on one side and PDMS posts (25 μm diameter and 15 μm spacing) on the other side, creating spatial field nonuniformities across the channel cross-section orthogonal to sample flow. The device was fabricated in a single layer to a depth of 50 μm using SU8 photolithography on a 4-inch silicon wafer. The silicon wafers were then silanized to allow for subsequent demolding of PDMS. PDMS (Dow Chemicals) was poured onto the silicon master for micromolding at a ratio of 10:1 (base to cross-linker) and allowed to cross-link at 70 °C for 12 h. Devices were then punched to create inlets and outlets for the electrode, sample, and sheath channels. Separately, electrodes for impedance cytometry were patterned on a glass wafer (University Wafer) using the lift-off technique, by patterning the AZ1505 resist (MicroChemicals GmbH) and electron beam deposition (Denton Vacuum) of metals (100 Å Ti adhesion layer and 1000 Å Au layer). The metal-patterned glass wafer was laser cut (VSL 3.5, Universal Laser Systems, Scottsdale, AZ). The PDMS channel features and the coverslip glass chip were activated using Oxygen plasma (Tergeo, PIE Scientific), with channel features for impedance cytometry aligned to the coplanar electrodes on the glass chip per Figure 1A using a stereo microscope and then put in contact for bonding. The electrode channels for DEP excitation were then filled with liquefied Field’s metal, as described previously.^44,48^ Briefly, the device was immersed in a water bath at 65 °C, and the liquefied Field’s metal (RotoMetals) was introduced through a syringe using positive pressure. After complete filling of the electrode channel, the device was allowed to cool at room temperature, resulting in solidification of the metal.

### Device Operation

Prior to introducing the sample and sheath flows for focusing the cell sample, the sample inlet and outlets were filled with 3% BSA (in 1x PBS) and left at room temperature for 30 min. The BSA solution was then removed. Sample and sheath fluid flows were introduced into their respective inlets using syringe pumps (Cetoni GmbH). Net flow rates of 3.6 μL/min were used for pDEP enrichment, based on a cell sample flow rate of 0.6 μL/min, sheath flow near the orifice edge at 0.9 μL/min, and sheath flow near the posts at 2.1 μL/min. The assembled chip was placed on a microscope stage equipped with a CMOS camera (Hamamatsu) for imaging cell streamlines. The function generator and amplifier system from the CytoR1 instrument (CytoRecovery, Blacksburg, MD) were connected to electrodes on the microchip to deliver the requisite voltage and frequency for dielectrophoretic separation. On-chip impedance cytometry (Figure 1C) was conducted in each outlet channel of the microfluidic device (detection region ∼50 μm depth by ∼50 μm width) at a total flow rate of 3.6 μL min^–1^ (neMESYS, Cetoni) in media used for dielectrophoretic separations. AC signals for impedance acquisition were optimized for this medium using frequencies (0.5–0.05 MHz) and voltages (2–10 V_pp_), applied to the central electrode of the coplanar three-electrode assembly. An impedance spectroscope (HF2IS, Zurich Instruments) was used, and the current signal at the adjoining side electrodes was acquired (sample rate = 1.4 × 10^4^ samples s^–1^ and converted using a current amplifier (HF2TA, Zurich Instruments). Lock-in amplification (HF2TA, Zurich Instruments) was used to separate the real and imaginary signal components at each frequency to compute the impedance magnitude and phase (Supporting Information, Section B). The impedance signal data (|Z| and ϕZ) were thresholded using MATLAB code to trigger the DEP amplifier from its serial port, so that the DEP excitation voltage and frequency could be varied to optimize for live cell numbers of specific size ranges in the pDEP-separated fraction. This closed-loop operation for impedance-based monitoring for the target cell type in the separated fraction enabled optimization of the DEP separation conditions to maximize collection efficiency and sample purity from heterogeneous and unknown starting samples.

### Flow Cytometry

The untreated, drug-treated, and mixed samples of GFP-expressing PDAC cells were assessed using a Beckman Cytoflex flow cytometer, and the data were analyzed using Beckman CytExpert Software. The samples were centrifuged and washed twice before analysis. Cells were gated based on Forward (FSC) and Side Scatter (SSC) to exclude debris (Figure S6). To exclude doublets, single cells were gated based on Side Scatter–Area vs Height. Live–dead assays were based on staining using APC Annexin V (Biolegend) and Zombie NIR or ZNIR (Biolegend) to quantify the viability and apoptotic levels. The intrinsic GFP signal was calibrated against ZNIR-stained cells for using GFP to measure the viability.

### COMSOL Simulation

Electrical field screening simulation for coplanar electrodes in the microchannel to measure impedance signal shapes and current conduction at each frequency of interest was conducted using the electric current module, flow module and particle tracing modules of COMSOL software, as per our previous work.^51,52^

### Data Analysis

Impedance cytometry data were processed and analyzed using custom code written in MATLAB (R2024a, MathWorks). The impedance signal was normalized against the frequency-independent impedance response of the reference polystyrene beads by dividing the impedance data by the mean impedance data of the reference beads. Due to normalization, the impedance phase is herein reported in arbitrary units. Normalized impedance magnitude is used to compute the metric of electrical diameter by calculating $\sqrt[{3}]{{|Z|}_{0.05\mathrm{MHz}}}$ and using the polystyrene beads for size reference. The definition of the gates for cell phenotypic classification is based on ∼1000 events for each sample type.

### Statistical Analysis

These were performed on processed data sets from three independent samples using MATLAB R2024a to distinguish significant differences between the various drug treatment and separation conditions.

## Supplementary Material





## Notes

This includes evidence of viscoelastic focusing (Figure S1), DEP-induced impedance signal disruption (Figure S2), electric field and impedance simulations (Figure S3), correlation of cell electrical size to physical size (Figure S4), absence of cell viability loss after passage through the device (Figure S5), and flow cytometry validation (Figure S6), as well as fitting parameters for simulating cell dielectric dispersions (Table S1) along with the dielectric shell model (Figure S7) (PDF)
Movie M1 of cell capture at electrodes (AVI)
