# Supplementary material for: On-Chip Integration of Impedance Cytometry for Inline Optimization of Dielectrophoretic Separations on Multiple Cellular Biophysical Metrics
Source: ACS Sens. 2025 Jun 6;10(6):4116–26. doi: 10.1021/acssensors.5c00192 (PMC12210248; doi:10.1021/acssensors.5c00192)
Supplement: Supplementary file 1 [file se5c00192_si_001.pdf]

Supporting Information

For

"On-chip integration of impedance cytometry for inline optimization of dielectrophoretic separations on multiple cellular biophysical metrics"

Javad Jarmoshti[a], Abdullah-Bin Siddique[a], Aditya Rane[b], Alexandra R. Hyler[c], Sara Adair[d], Todd W. Bauer[d], Nathan S. Swami [a,b,*]

a – Electrical and Computer Engineering, University of Virginia, Charlottesville, Virginia 22904, USA

b – Chemistry, University of Virginia, Charlottesville, Virginia 22904, USA

c – CytoRecovery, Inc., Blacksburg, Virginia 24060, USA

d –Surgery, School of Medicine, University of Virginia, Charlottesville, Virginia 22904, USA

Supplementary Figures

Figure S1. Viscoelastic focusing validated by electric field screening & measurements

Figure S2. Disruption of impedance signals due to cell capture at the acquisition electrodes by dielectrophoresis

Figure S3. Using electric field screening simulations to compute impedance signals

Figure S4. Correlation of electrical size from impedance cytometry to physical size of corresponding cells from image flow cytometry

Figure S5. Confirming the absence of cell viability loss based on GFP signal in optimal media (1x PBS on left) versus (right) after passage through device in DEP buffer

Figure S6. Flow cytometry for size-based gating of live vs. dead cells in the: A. Input; B. noDEP; and C. pDEP outlets shows the absence of cell debris and dead cells in pDEP outlet.

Figure S7. Dielectric shell model of cell based on Maxwell's mixture theory

Supplementary Table

Table S1: Fitting parameters for simulating cell dielectric dispersions

Supplementary Movie

Movie M1. Cell capture at the acquisition electrodes by dielectrophoresis in media of low conductivity (55 µS/cm) at high acquisition voltages (10 V_{pp}) and low frequencies (50 kHz)

*All correspondence should be addressed.

A. Supplementary Results

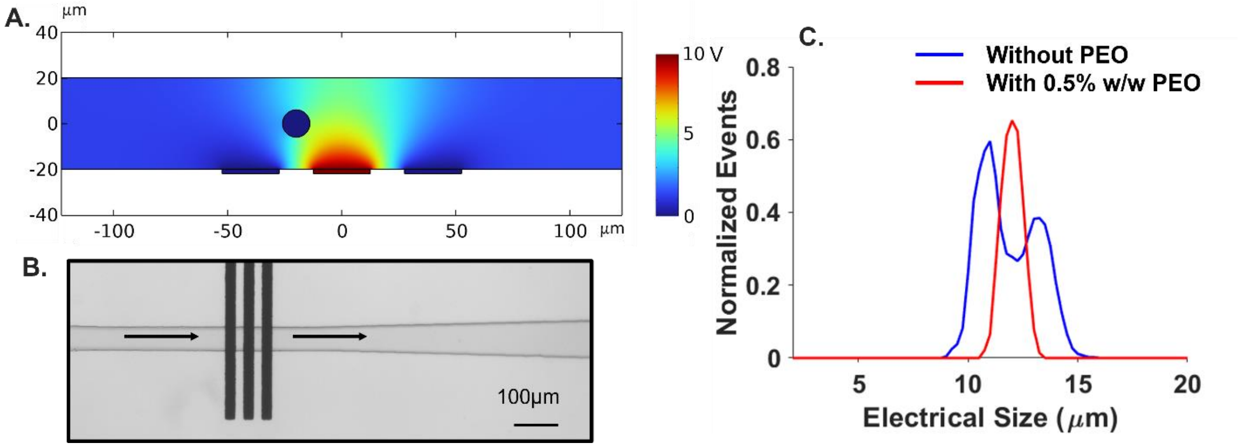

Figure S1: A. Electric field simulations of coplanar electrodes in the microchannel to determine sensitivity of impedance cytometry signals to media conductivity, as reported in **Fig. 2B** of manuscript. B. Set-up for impedance cytometry measurements downstream of dielectrophoretic separations, as reported in **Fig. 2C** of the manuscript. C. Impedance-based electrical size ($\sqrt[3]{|Z|_{0.05\,\text{MHz}}}$) of 12 µm polystyrene beads in media of low conductivity (σ_{med} of 55 µS/cm) measured without versus with PEO (polyethylene oxide in 0.5% w/w of 0.6 MDa) to validate elasto-inertial focusing. In absence of PEO, two distinct data clusters representing cell focusing close to electrode and away from electrode are apparent, whereas in the presence of PEO at 10 µL/min flow rate, a single data cluster representing elasto-inertial focusing of beads at the center of channel depth is apparent.

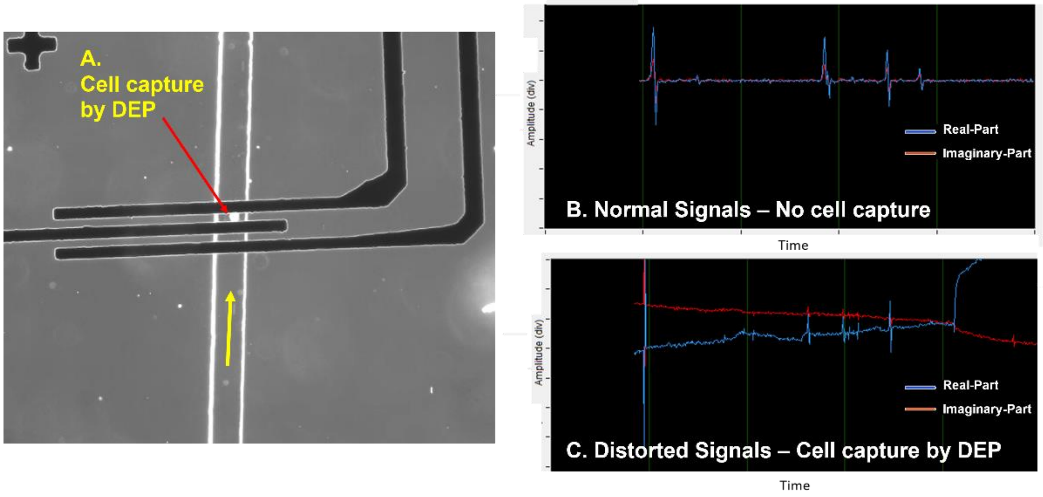

Figure S2: A. Cell capture at the impedance acquisition electrodes due to dielectrophoresis or DEP (see arrow of trapped cell) causes disruption of the background of impedance cytometry signals, as apparent from: B. Normal signals (no cell capture), versus C. distorted signals due to shifted background by DEP cell capture (also see movie M1 of cell capture).

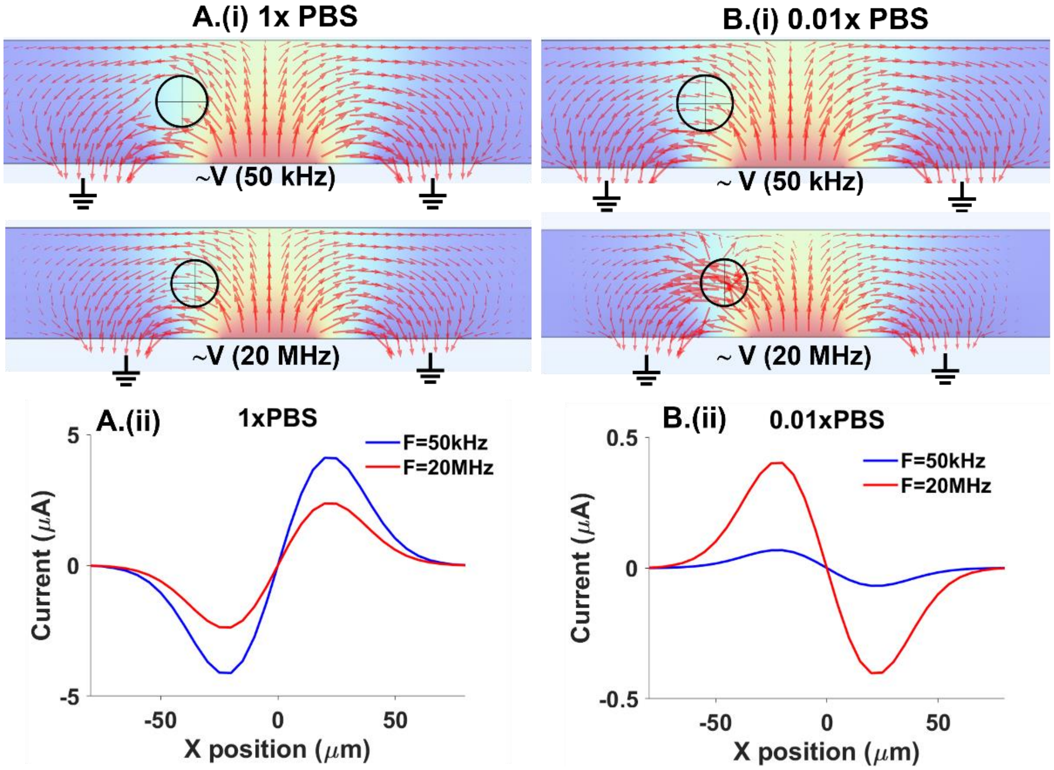

Figure S3: Using electric field screening simulations (top row (i)), the respective impedance signals (bottom row (ii)) are computed at the acquisition frequencies of 50 kHz (blue curve) and 20 MHz (red curve) using: **A.** 1x PBS (σ_{med} = 16000 µS/cm); and **B.** 0.01x PBS (σ_{med} = 160 µS/cm) for live PDAC cells per dielectric model in **Supplementary Methods Section B**. **B(ii)**: In 1x PBS (high σ_{med}), the current is highly screened at the cell exterior at 50 kHz and screened to a less degree at 20 MHz (per electric field simulations in **A.(i))** causing current signals that are high for the blue curve at 50 kHz due to $\sigma_{cell} << \sigma_{med}$ and lower for the red curve at 20 MHz due to $\sigma_{cell} < \sigma_{med}$, thereby starting at a negative signal level due to polarization in the media interface to the cell. **B(ii)**: In 0.01x PBS (low σ_{med}), on the other hand, the cell interior is more conductive than the exterior media, thereby causing polarization and more electric field lines to pass through the cell interior at 50 kHz and 20 MHz (per electric field simulation in **B.(i))**. This leads to a reversal in the differential current profile (per bipolar Gaussian shape in **B(ii)** ***vs.*** **A(ii)**) that shows some field penetration at 50 kHz (low positive signal) and greater field penetration at 20 MHz (higher positive signal). It is noteworthy that the simulated impedance signal shape at 50 kHz for live PDAC cell in low σ_{med} (B(ii)) resembles that obtained for measurements of live PDAC cells (Fig. 3B(iii) of manuscript) after normalization of impedance phase (ϕZ) signals of polystyrene beads to ϕZ=0. As a result, live cells show positive ϕZ and dead cells show negative ϕZ levels, per the data with PDAC samples in **Fig. 3C**.

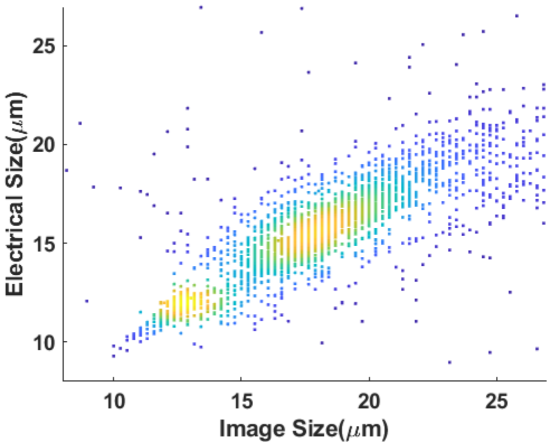

Figure S4. The electrical size of PDAC cells (determined from $\sqrt[3]{|Z|_{0.05\ \mathrm{MHz}}}$ after normalization to 12 μm polystyrene beads) is compared to the physical size of corresponding cells determined by high-speed imaging. The plot shows a high degree of correlation.

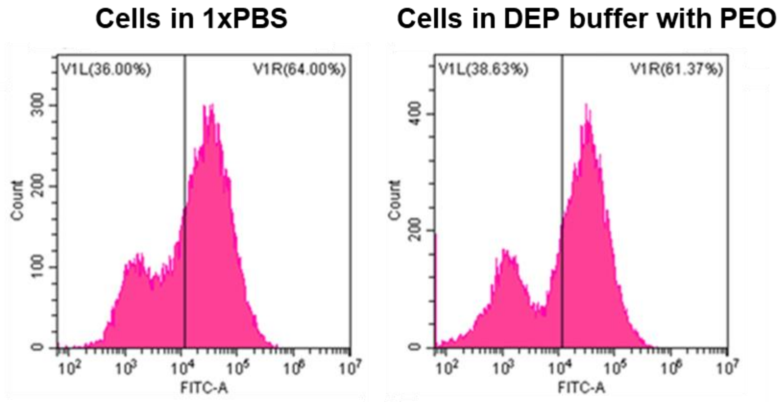

Figure S5: Confirming the absence of cell viability loss based on GFP signal (correlated to live-dead assay in **Fig. 4B**) in optimal media (1x PBS on left) versus (right) after passage through device in DEP buffer (sucrose and BSA or Bovine Serum Albumin in 0.01x PBS adjusted to media conductivity of 55-220 μS/cm) with 0.5% w/w of 0.6 MDa PEO for elasto-inertial focusing.

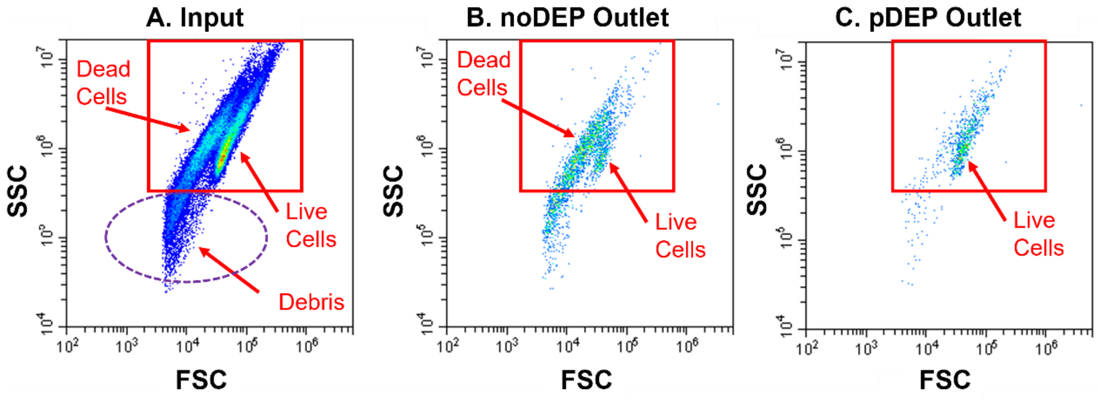

Figure S6: Flow cytometry for size-based gating of live vs. dead cells in the: A. Input; B. noDEP; and C. pDEP outlets shows the absence of cell debris and dead cells in pDEP outlet.

B. Supplementary Methods – Dielectric Shell Modelling and dielectrophoresis calculations

For a cell suspended in a dielectric medium, the dielectric properties of the suspension can be determined using Maxwell's Mixture theory (**Fig. S7**) by calculating the complex permittivity of the suspension ($\tilde{\varepsilon}_{mix}$). To calculate the dielectric properties of the suspended cell, MMT-based shell models can be used. For the sake of simplification, the cell is modelled as a series of concentric shells, each with its own defined dielectric properties. The simplest model of a cell, a single shell model has two dispersions at its interfaces (medium-membrane and membrane-interior). The complex permittivity of the suspension ($\tilde{\varepsilon}_{mix}$) is:

$$\tilde{\varepsilon}_{mix} = \tilde{\varepsilon}_{medium}\frac{1 + 2\varphi f_{CM}}{1 - \varphi f_{CM}} \qquad (S1)$$

where $\tilde{\varepsilon}_{medium}$ is the complex permittivity of the surrounding medium, φ is the volume fraction of the particle in the medium and f_{CM} is the Clausius-Mossotti factor of the cell in the mixture. $\tilde{\varepsilon}$ can be defined as:

$$\tilde{\varepsilon} = \varepsilon_0\varepsilon - j\frac{\sigma}{\omega} \qquad \text{(S2)}$$

where ε is the permittivity, ε_0 is the constant permittivity in vacuum, σ is the conductivity, ω is the frequency of the applied electric field and $j^2 = -1$.

For a shell model, the Claussius-Mosotti factor of the cell in the mixture (f_{CM}) is given by:

$$f_{CM} = \frac{\tilde{\varepsilon}_{cell} - \tilde{\varepsilon}_{medium}}{\tilde{\varepsilon}_{cell} + 2\tilde{\varepsilon}_{medium}} \qquad (S3)$$

The complex permittivity of the cell ($\tilde{\varepsilon}_{cell}$) in a single shell model can be modelled as:

$$\tilde{\varepsilon}_{cell} = \tilde{\varepsilon}_{membrane}\frac{\gamma^3 + 2\left(\frac{\tilde{\varepsilon}_{interior} - \tilde{\varepsilon}_{membrane}}{\tilde{\varepsilon}_{interior} + 2\tilde{\varepsilon}_{membrane}}\right)}{\gamma^3 - \left(\frac{\tilde{\varepsilon}_{interior} - \tilde{\varepsilon}_{membrane}}{\tilde{\varepsilon}_{interior} + 2\tilde{\varepsilon}_{membrane}}\right)} \qquad (S4)$$

with;

$$\gamma = \frac{r_{cell}}{r_{cell} - d_{membrane}} \qquad (S5)$$

where r_{cell} is the radius of the cell and $d_{membrane}$ is the thickness of the cell membrane. With the calculation of the complex permittivity of the suspension ($\tilde{\varepsilon}_{mix}$), the impedance of the mixture ($\tilde{Z}_{mix}$) can be calculated as:

$$\tilde{Z}_{mix} = \frac{1}{j\omega\tilde{\varepsilon}_{mix}G} \qquad (S6)$$

where G is the geometric constant of the system, and can be approximated as:

$$A_{electrode}/d_{electrode} \qquad (S7)$$

where $A_{electrode}$ is the surface area of the electrode and $d_{electrode}$ is the distance between the electrodes. Since $\tilde{Z}_{mix}$ is frequency dependent, relaxation curves for impedance magnitude ($|Z|$) and phase (ϕZ) can be calculated using:

$$|Z| = \sqrt{Re(\tilde{Z}_{mix})^2 + Im(\tilde{Z}_{mix})^2} \qquad \text{(S8)}$$

$$\phi Z = \tan^{-1}\frac{Im(\tilde{Z}_{mix})}{Re(\tilde{Z}_{mix})} \qquad (S9)$$

where $Re(\tilde{Z}_{mix})$ and $Im((\tilde{Z}_{mix})$ are the real and imaginary parts of the complex impedance of the mixture $(\tilde{Z}_{mix})$.

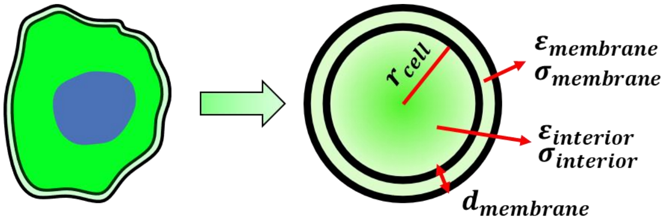

Fig S7: Single shell model of cell based on Maxwell's mixture theory.

Table S1: Fitting parameters for simulating cell dielectric dispersions

Parameter	Value for live cell	Value for dead cell
Vacuum permittivity (ε_0)	8.85×10^{-12} F m^{-1}	8.85×10^{-12} F m^{-1}
Medium conductivity (σ_{medium})	1.6 S/m & 0.016 S/m	1.6 S/m & 0.016 S/m
Medium permittivity (ε_{medium})	80	80
Membrane Conductivity ($\sigma_{membrane}$)	10^{-6}S/m	10^{-2} S/m
Membrane Permittivity ($\varepsilon_{membrane}$)	5.87	20
Interior Conductivity ($\sigma_{interior}$)	0.5 S/m	0.01 S/m
Interior Permittivity ($\varepsilon_{interior}$)	60	70
Cell Radius (r_{cell})	6 μm	6 μm
Membrane Thickness ($d_{membrane}$)	14 nm	14 nm
Surface Area of electrode ($A_{electrode}$)	10^{-9} m^2	10^{-9} m^2
Distance between electrodes ($d_{electrode}$)	60×10^{-6} m	60×10^{-6} m

Based on this, the dielectrophoretic force (F_{DEP}) experienced by the cells due to their polarization in a non-uniform electric field can be calculated as:

$$F_{DEP} = 2\pi\varepsilon_{medium}{r_{cell}}^3 Re(f_{CM})(\nabla E^2) \qquad \text{(S10)}$$

where ∇E^2 is the Laplacian operator of the applied electric field squared. The DEP mobility (μ_{DEP}) is lowered with increasing media viscosity (η), thereby requiring greater ∇E^2 for equivalent velocities to cells under the balance of DEP and viscoelastic drag forces (V_{DEP}), which is addressed in our work by 6-8-fold higher voltages for effective pDEP deflection of cells in viscoelastic media (0.5% w/w of 0.6 MDa PEO of ~8-fold higher viscosities).

$$V_{DEP} = \mu_{DEP}(\nabla E^2) \text{ where } \mu_{DEP} = \frac{{r_{cell}}^2\varepsilon_{medium}}{3\eta} Re(f_{CM}) \qquad \text{(S11)}$$
